# Advancing autologous urothelial micrografting and composite tubular grafts for future single-staged urogenital reconstructions

**DOI:** 10.1038/s41598-023-42092-3

**Published:** 2023-09-20

**Authors:** Nikolai Juul, Fatemeh Ajalloueian, Oliver Willacy, Clara Ibel Chamorro, Magdalena Fossum

**Affiliations:** 1 Laboratory of Tissue Engineering, Rigshospitalet, Faculty of Health and Medical Sciences, University of Copenhagen, Copenhagen, Denmark; 2 Division of Pediatric Surgery, Department of Surgery and Transplantation, Copenhagen University Hospital Rigshospitalet, Copenhagen, Denmark; 3 Department of Health Technology, Technical University of Denmark, Kgs. Lyngby, Denmark; 4 Laboratory of Tissue Engineering, Department of Women’s and Children’s Health, Karolinska Institutet, Stockholm, Sweden

**Keywords:** Preclinical research, Tissue engineering

## Abstract

Urogenital reconstructive surgery can be impeded by lack of tissue. Further developments within the discipline of tissue engineering may be part of a solution to improve clinical outcomes. In this study, we aimed to design an accessible and easily assembled tubular graft with autologous tissue, which could be constructed and implanted as a single-staged surgical procedure within the premises of an ordinary operating room. The ultimate goals would be to optimize current treatment-options for long-term urinary diversion. Therefore, we evaluated the optimal composition of a collagen-based scaffold with urothelial micrografts in vitro, and followingly implanted the construct in vivo as a bladder conduit. The scaffold was evaluated in relation to cell regeneration, permeability, and biomechanical properties. After establishing an optimized scaffold in vitro, consisting of high-density collagen with submerged autologous micrografts and reinforced with a mesh and stent, the construct was successfully implanted in an in vivo minipig model. The construct assemblance and surgical implantation proved feasible within the timeframe of a routine surgical intervention, and the animal quickly recovered postoperatively. Three weeks post-implantation, the conduit demonstrated good host-integration with a multilayered luminal urothelium. Our findings have encouraged us to support its use in more extensive preclinical large-animal studies.

## Introduction

Reconstructive surgery of the urinary system, following either trauma, cancer treatment, or congenital malformations, has traditionally relied largely on the reuse of native tissue harvested elsewhere in the body. Most often, gastrointestinal tissues have been used to reconstruct the bladder or the catheterizable bladder conduits, unfortunately often at the price of several adverse side effects such as chronic infections, tissue stenosis, bladder stone formation, electrolyte derangement, and even cancer^1–5^. In certain congenital or acquired conditions, a long-term need for urinary diversion is needed to avoid urinary stasis and irreversible damage to the kidneys. In these cases, the more frequent strategy is to transpose the cecal appendix to form an anastomosis between the bladder and the skin, hereby utilizing the appendix as a conduit for urinary diversion (an appendicovesicostomy). The surgery requires intraperitoneal dissection with a subsequent risk of adhesions and complications hereof, and is often associated with postoperative stomal stenosis^6–8^. Tissue engineering holds a potential of aiding surgical treatments, by providing scaffolds for organ reconstruction which are customizable and attain favorable compatibility profiles^9,10^. However, among previous tissue-engineered urinary models, it has been widely suggested that acellular scaffolds are more prone to scar- and stricture formation in vivo^11,12^. Furthermore, the scaffold needs to provide favorable biomechanical properties, similar to those of native bladder or urethral tissue, during wound healing and before complete biodegradation^13^.

In this study, we designed a tubular collagen-based compressed scaffold seeded with porcine urothelial tissue micrografts and reinforced with a surgical mesh and a luminal stent, as an alternative tissue-engineered urinary bladder conduit. Our group has previously presented the perioperative layered autologous tissue expansion graft (PLATE graft), which is a collagen and biomaterial reinforced graft for epithelial organ tissue expansion^14^. This study is a continuation of the methodology, where the specific graft components have been evaluated against various biomaterials^10,14–16^. Our primary aim was to construct a tubular conduit with commonly available composites, favorable physiological properties, and the possibility of assembling the construct as a single-staged procedure during the primary reconstructive surgery. To assess the optimal properties of the conduit, we compared two collagen compositions, micrograft positioning, and mesh fiber orientation during a series of in vitro experiments, evaluating both histological regeneration, macromolecular permeability, and biomechanical performance. We further optimized the methodology for tubular tissue engineering by introducing a biodegradable stent for structural integrity. The assessment of in vitro degradation of the scaffold served in this study as a proxy for the expected impacts from in vivo implantation, further justifying live animal involvement. After establishing a feasible model in vitro, we proceeded with an in vivo model, implanting our tubular construct, embedded with autologous urothelial micrografts, in an in vivo minipig model. We finally assessed the technical feasibility of the procedure, any short-term potential adverse host reactions, macroscopic appearance of the conduit and tissue regeneration by histology, 3 weeks post-implantation. Tissue engineering methods for fabrication of tubular scaffolds include casting, electrospinning, rolling, 3D printing, and decellularization. However, a secondary cell-seeding step is often required, hindering the clinical translation of the concepts^17^. Among the advancements presented within this paper, the application of a PLATE graft for complete tubular organ reconstruction is a key technical progression of the methodology, with future potential for clinical translation.

## Results

### In vitro optimization of a collagen-based micrografted scaffold

**Figure 1:**
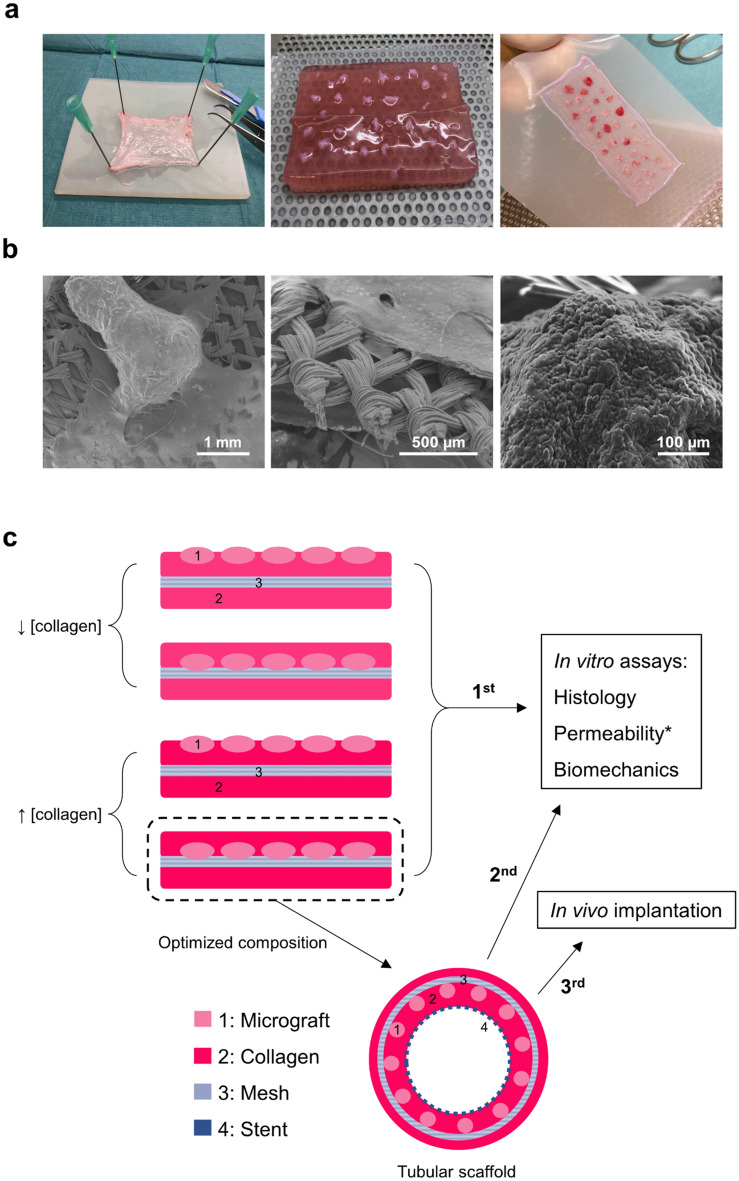
In vitro study design and basic scaffold composition. (**a**) Sequential representation of the dissection of porcine bladder urothelium (left), minced micrografts placed on top of the mesh-reinforced collagen gel before compression (middle), and after compression (right). (**b**) Scanning electron microscopy (SEM) images of a urothelial micrograft on top of collagen and mesh (left), close-up image of the collagen-embedded mesh directly after compression (middle), and urothelial cells migrating from the micrograft onto the scaffold after 2 weeks in culture (right). (**c**) A schematic representation of the initial study conditions evaluated to assess the optimal scaffold composition, and the sequential order of experiments performed. Scaffolds with either high- or lower density collagen, and with micrografts either on top or submerged in the collagen, were compared in vitro during a 1-month study period and evaluated histologically and in permeability and biomechanical assays. The optimized scaffold composition was used to construct a tubular scaffold which was subjected to similar in vitro evaluation after 1 month in culture (except for permeability assessment, marked with *). Finally, an identical tubular scaffold with autologous micrografts was surgically implanted in a female minipig and assessed 3 weeks after implantation.

Initially, we performed in vitro evaluations of compressed, non-tubularized collagen scaffolds, reinforced with a mesh and seeded with urothelial micrografts dissected from the bladder mucosa of three Landrace pigs (Fig. 1a,b). We compared two different collagen concentrations and two different micrograft locations during a 4-week incubation period. Based on our assessments of the four initial study conditions, the optimal composition was then applied for in vitro evaluation in a tubularized form and finally implanted in vivo in a minipig model (Fig. 1c).

**Figure 2:**
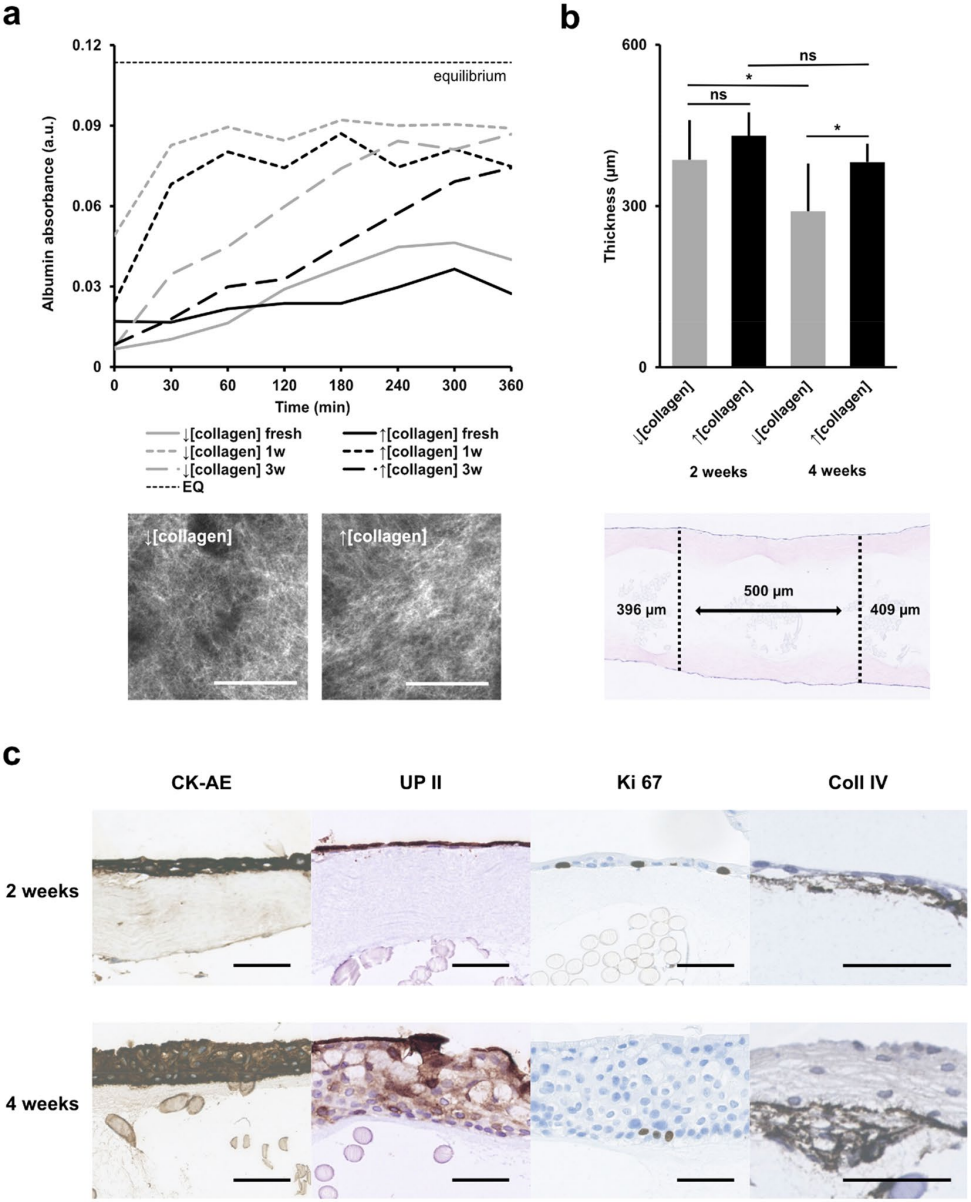
Variations of collagen concentration and the in vitro effect on permeability and scaffold thickness over time. (**a**) Albumin absorbance over time (absorbance units) measured from the receptor solution, with cellularized scaffolds of either higher or lower collagen density at different incubation times used to separate the donor solution. Equilibrium concentration between donor and receptor solutions indicated for reference (dotted line). Confocal reflectance microscopy of the top collagen layers in compressed sheets with either lower or higher collagen density (bottom). All scale bars are 50 µm. (**b**) Scaffold thickness after compression and with relative differences between different collagen densities at two timepoints. Example of scaffold thickness measurements after 2 weeks in culture (bottom). (**c**) Immunohistochemical stains of the micrografted scaffold after two and 4 weeks in culture, illustrating epithelial stratification (pancytokeratin AE), cell differentiation (uroplakin II), cell proliferation (ki67), and basement membrane formation (collagen IV). All scale bars mark 50 µm, unless otherwise stated.

At the beginning of the experiment, we evaluated albumin permeability across freshly compressed collagen scaffolds, still not covered with urothelial cells. Collagen scaffolds with a higher concentration of collagen solution (80%) were less permeable to albumin than scaffolds with a lower collagen concentration (60%), indicating a denser collagen network. Both collagen densities presented with an increased permeability after the first week in culture, indicating changes in the collagen composition in vitro. After 3 weeks in culture, the permeability of the scaffolds had decreased markedly again, likely due to the formation of a well-defined urothelium with barrier function (Fig. 2a). After 2 weeks in culture, the scaffold thicknesses of the two collagen densities did not differ significantly (386 vs. 431 µm, *p* = 0.1288). From 2–4 weeks in culture, the thickness of the lower density scaffolds had significantly decreased (386 vs. 290 µm, *p* < 0.05), whereas the thickness of the higher density scaffold had not significantly decreased over time. After 4 weeks, the higher density scaffolds were significantly thicker than the lower density scaffolds (382 µm vs. 290 µm, *p* < 0.05) (Fig. 2b). In both collagen constructs (higher and lower collagen densities), a well-defined mono- or multilayered (2–6 cell layers) proliferative urothelium was covering most of the scaffold after 2 weeks. Furthermore, after 2 weeks, deposited collagen IV was visible under the urothelium in both groups, indicating formation of a basement membrane. After 4 weeks, several areas of urothelium had further stratified and was expressing abundant levels of uroplakin II, indicating urothelial cell differentiation (Fig. 2c).

**Figure 3:**
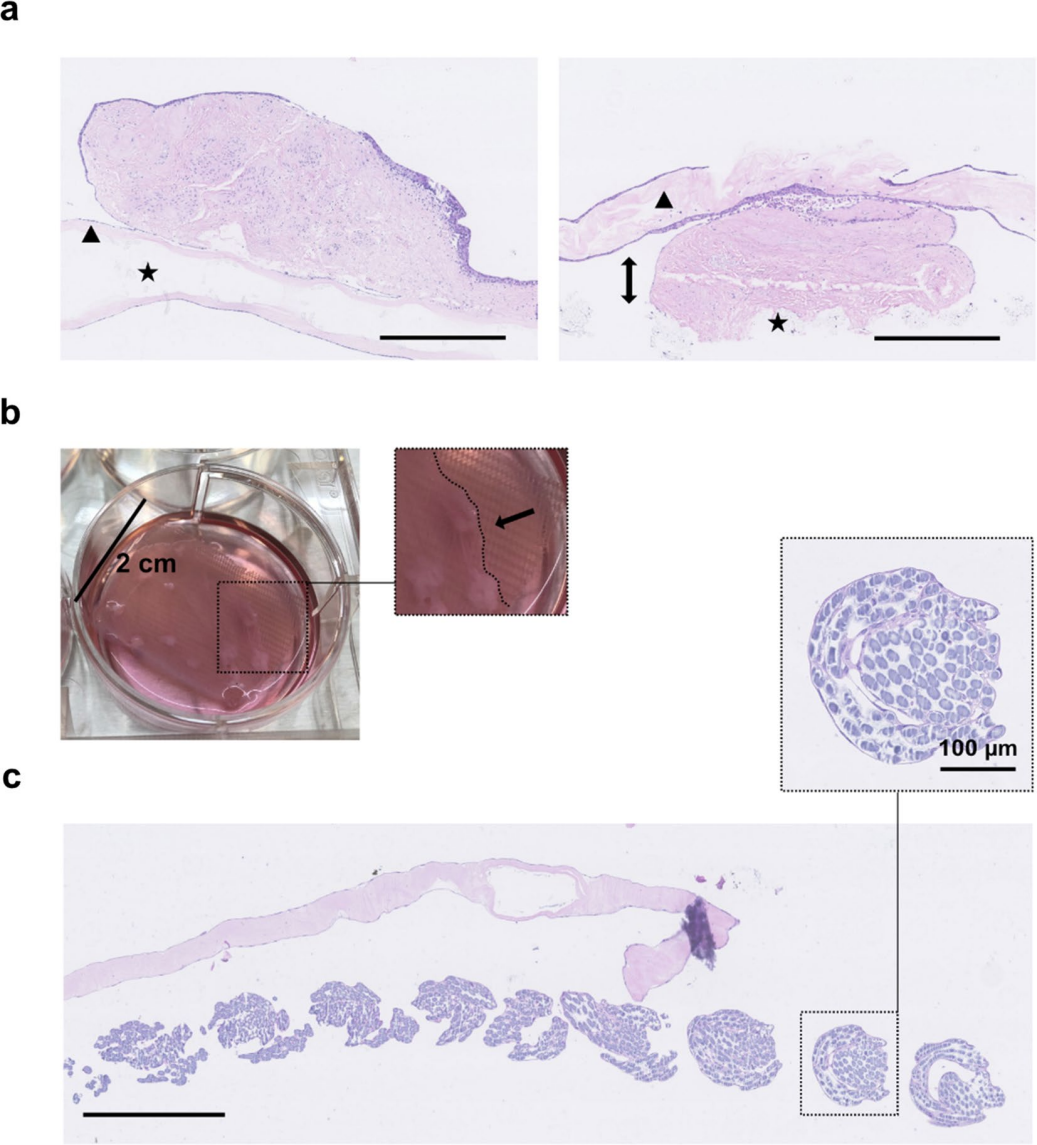
Micrograft location within the collagen scaffold in vitro. (**a**) Example of a collagen scaffold with micrografts placed on top of the collagen layers (triangle), with embedded mesh (star)(left), and an example of a collagen scaffold with micrografts placed beneath the top collagen layer with markings of the gaps between collagen layers adjacent to micrografts (double arrows). (**b**) Macroscopical image of a partially delaminated low-density collagen scaffold with micrografts on top after 4 weeks incubation. (**c**) Microscopical image from the same delaminated sample with H&E stains, showing cell infiltration of the mesh fibers. HE stains. All scale bars are 500 µm, unless otherwise noted.

When comparing the effect of positioning micrografts either on top or submerged below the collagen (directly on the mesh), both groups were equally covered with proliferative cell layers after 2 weeks in culture, indicating that the embedding of micrografts within the collagen did not limit cellular mobility and viability (Fig. 3a). This encouraged us to continue the complete embedding of micrografts, in later experiments, since the scaffold bio-adhesion would intuitively improve. A minor gap between the mesh and collagen was visible adjacent to the micrografts placed directly on the mesh, although this did not result in any collagen delamination (i.e., collagen sliding off the mesh). After 4 weeks, partial areas of collagen delamination were observed samples of both lower and higher collagen density (33% and 11% of the samples, respectively), whereas the delamination was not related to the micrograft positioning (i.e., on top or below collagen) (Fig. 3b). In the areas of partial delamination, the mesh had been readily infiltrated with urothelial cells, indicating that the material itself served as a viable platform for the cells to proliferate on (Fig. 3c). Based on these findings, we concluded that a higher collagen density provided a better barrier function, and that submerged micrografts improved the tissue adhesion without damaging the cells.

### Scaffolds seeded with human urothelial cells from bladder-washings

**Figure 4:**
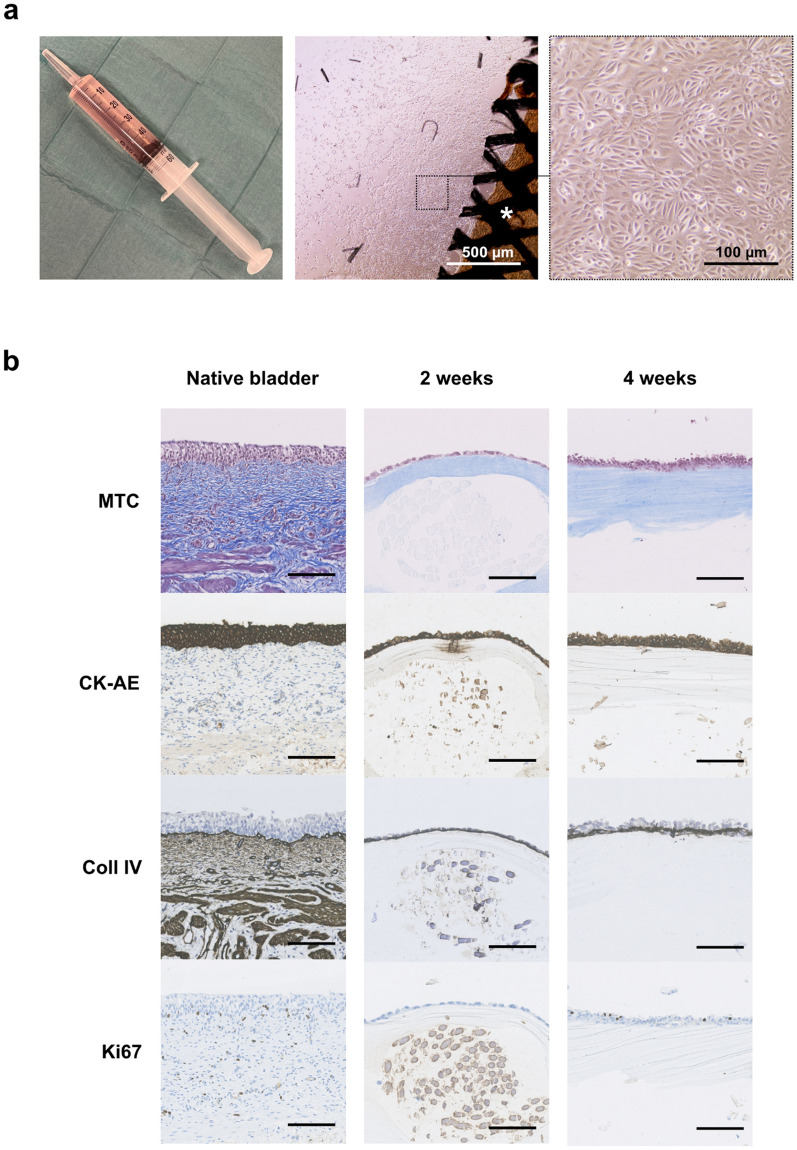
In vitro culturing of human urothelial cells from bladder-washing. (**a**) A 50 mL syringe used for collecting bladder-washed saline at the operating room (left), confluent cells in culture 3 weeks after seeding(middle), and cells migrating off the sides of a mesh-reinforced collagen scaffold 1 week after seeding (right). (**b**) Histological sections of mesh-reinforced collagen scaffolds with bladder-washed urothelial cells seeded on top after two (middle column) and 4 weeks (right column) in culture and native porcine bladder wall as a reference (left column). Masson’s trichrome (MTC) to visualize the collagen layers, Cytokeratin AE1/3 (CK-AE) to identify epithelial phenotype, collagen IV (coll IV) staining to identify the basement membrane, and ki-67 as a proliferative marker. All scale bars mark 200 µm.

We furthermore evaluated if human cells were able to proliferate on the scaffold. For this purpose, human urothelial cells were successfully obtained from bladder barbotage performed at the clinical department. After further propagation in culture flasks, a cell suspension was seeded directly on top of the compressed collagen scaffold (Fig. 4a). After 2 weeks, the urothelial cells covered the entire surface of the scaffold as a mono- or multilayered confluent epithelium. After 1 week in culture, collagen IV had been deposited by the cells, indicating the formation of a basal membrane. After 4 weeks in culture, the epithelium resembled the micro-anatomy of native bladder urothelial mucosa, in respect to larger multinucleated cells on top and proliferative cells lining the basal layers (Fig. 4b).

### In vitro construction and evaluation of a composite tubularized scaffold

**Figure 5:**
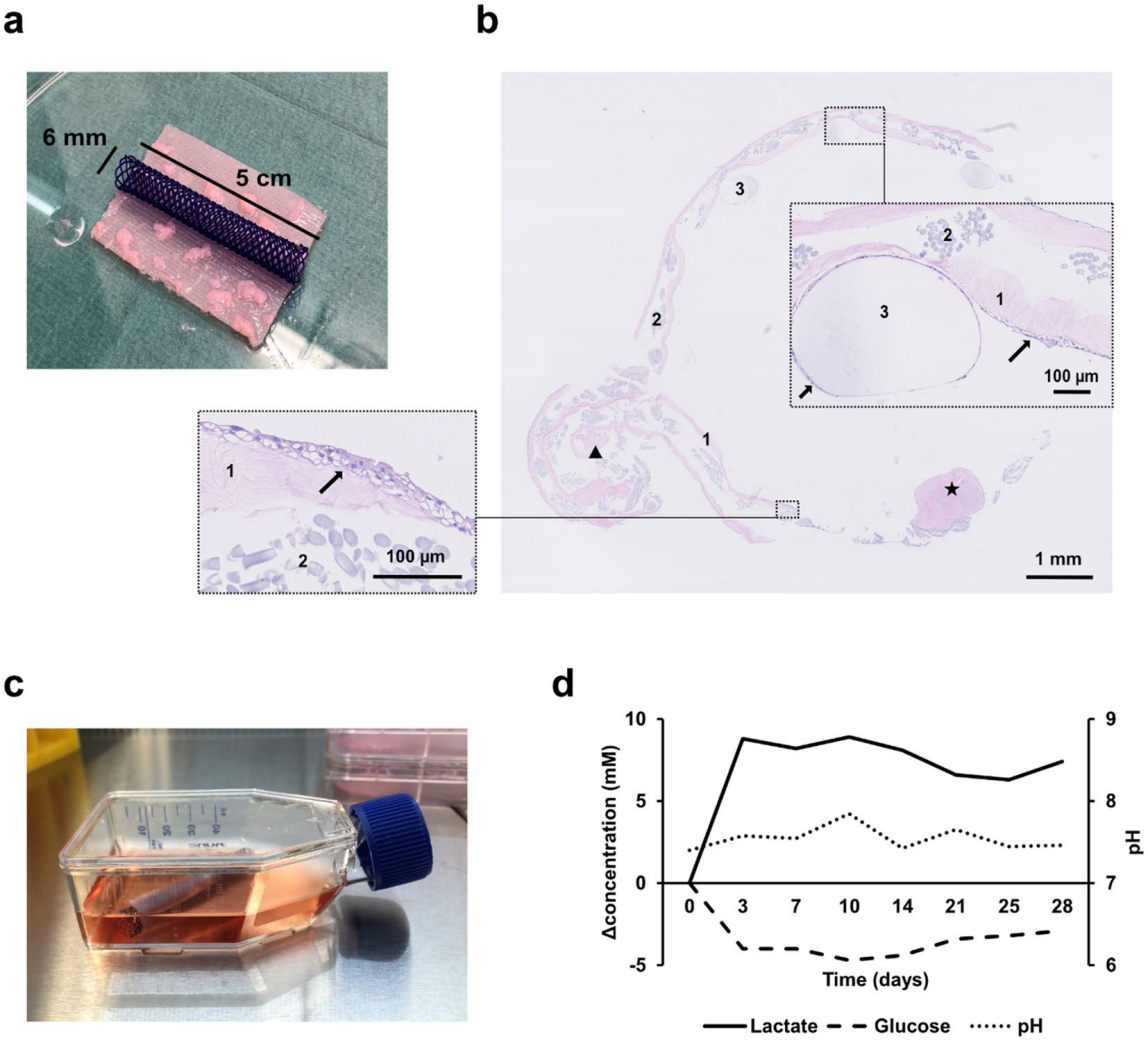
In vitro assessments of a tubularized scaffold. (**a**) A compressed high-density collagen scaffold, reinforce with mesh and with submerged micrografts, after compression and before suturing around a stent. (**b**) Cross-section of the scaffold after 4 weeks in culture. Markings indicate collagen layers (1), mesh fibers (2), stent remnants (3), micrografts (star), and suture line (triangle). In further magnified areas, urothelium is marked (arrows). HE staining. (**c**) Culture flask used for culturing the micrografted conduit. (**d**) Regularly measured levels of pH (left y-axis), lactate and glucose (right y-axis) from 24-h old culture medium and the change from the levels in fresh medium over 4 weeks (vertical axis).

A compressed high-density collagen-based scaffold with embedded porcine urothelial micrografts, reinforced with a knitted polyglactin mesh and sutured around a biodegradable stent, was cultured for 4 weeks in vitro in a regular culture flask (Fig. 5a,c). After 4 weeks in culture conditions, a multilayered urothelium covered the entire luminal circumference of the tubularized scaffold, including cell-colonization of the internal stent material (Fig. 5b). The levels of pH, lactate, and glucose, measured in 24-h-old culture medium, stabilized after three days, and remained within physiological limits throughout the study period, indicating that the degrading scaffold did not release significant amounts of acidic substances (Fig. 5d).

### In vitro biomechanical properties

**Figure 6:**
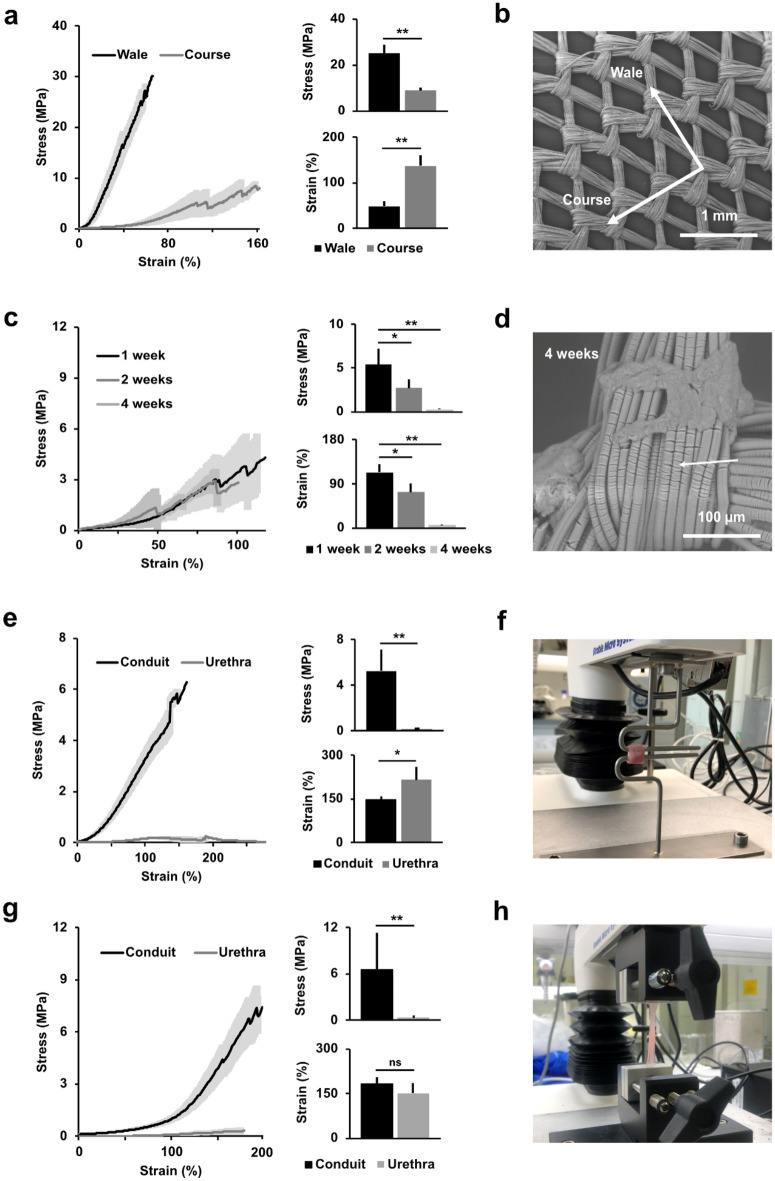
Biomechanical assessments of various study conditions in vitro. (**a**) Stress and strain curves depicting uniaxial tensile tests on knitted mesh in the course and wale directions (left) with maximum levels compared directly in bar charts (right). (**b**) Ultrastructural SEM image illustrating the different directions of the knitted Vicryl mesh. (**c**) Stress and strain curves depicting uniaxial tensile tests on knitted mesh at three timepoints (left) with maximum levels compared directly in bar charts (right). (**d**) Ultrastructural SEM image illustrating the degraded Vicryl mesh (arrow) after 4 weeks of incubation. (**e**) Stress and strain curves depicting circumferential hook tensile tests on sutured conduit (without the PDS stent) and native porcine urethra (left) with maximum levels compared directly in bar charts (right). (**f**) Hook test setup in process with mounted porcine urethra pulled circumferentially. (**g**) Stress and strain curves depicting uniaxial tensile tests on a sutured conduit (without the stent) and a native porcine urethra (left) with maximum levels compared directly in bar charts (right). (**h**) Uniaxial tensile test setup in process with mounted porcine urethra pulled longitudinally.

When comparing the two standard directions of the knitted mesh material, the course and the wale, in a uniaxial tensile test, we found that the wale direction pulled sizably larger stress levels than the course direction (25 vs. 9 MPa, *p* < 0.0001), whereas the course direction endured a higher strain (137% vs. 49%, *p* < 0.0001) (Fig. 6a–b). The mesh degraded in culture conditions with cells, and, from 1 week to 2 weeks in culture, the maximal stress levels in the course direction of the mesh reduced from 5 to 3 MPa, respectively (*p* < 0.05). Similarly, the maximum strain reduced from 113 to 73%, respectively (*p* < 0.05). After 4 weeks, the mesh could barely withstand minimal mechanical handling (mean stress 0.25 MPa and mean strain 6%), indicating almost complete in vitro degradation (Fig. 6c–d). When comparing a circumferential tension (wale mesh direction) of the sutured conduit (not including the luminal stent) to fresh native pig urethra, a significantly higher stress endurance was found in the conduit (5 MPa vs. 0.2 MPa, *p* < 0.0001), whereas the urethra endured a higher strain (216% vs. 148%, respectively, *p* < 0.05) (Fig. 6e–f). In a longitudinal tension (course mesh direction), the conduit also yielded higher average stress levels than the native urethra (6.5 MPa vs. 0.3 MPa, *p* < 0.0001), whereas the average strain levels were comparable between the conduit and the urethra (183% vs. 152%, *p* = 0.256) (Fig. 6g–h).

### In vivo surgical conduit implantation

**Figure 7:**
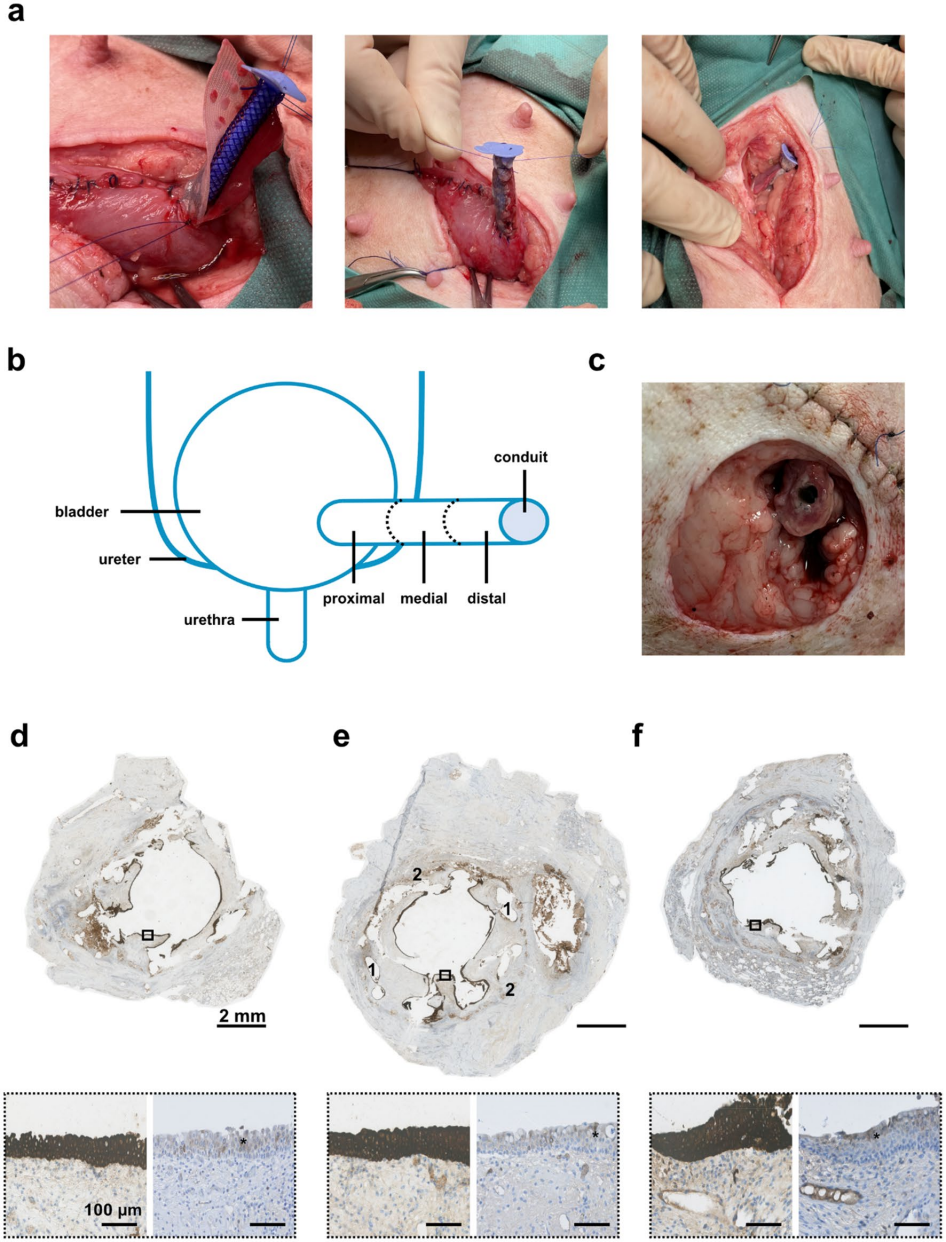
In vivo surgical implantation of the micrografted conduit. (**a**) The mesh-reinforced collagen scaffold with autologous urothelial micrografts being constructed, tubularized around the biodegradable stent and anastomosed to the bladder (left). An ACE stopper being fixed within the lumen of the conduit, and a ligature placed at the distal end to ensure patency (middle). The conduit then channeled through the abdominal muscle and placed subcutaneously, and non-absorbable suture-markings are placed at skin level (right). (**b**) Schematic illustration of the anatomical conduit implantation, and representation of the different conduit regions of interest. (**c**) The conduit dissected after 3 weeks with the ACE stopper removed. (**d**–**f**) Cross-sectioned specimen after 3 weeks in vivo from the proximal, medial, and distal conduit region, respectively, confirming luminal epithelialization (CK-AE stain), and magnified areas with confirmed differentiated urothelium (Uroplakin-II stain, right).

In continuation of the findings in our initial in vitro experiments, the conduit was successfully constructed and implanted in a pilot in vivo minipig model. The scaffold was constructed using the higher collagen density (80% collagen solution), with submerged urothelial micrografts, and the reinforcing mesh was applied with the course mesh-direction in the longitudinal axis of the conduit. The entire procedure, including perioperative construction of the conduit scaffold, lasted 3.5 h (Fig. 7a,b). The animal quickly recovered from the anesthesia and returned to its normal habitus. Normal food intake and urination was confirmed within 2 h postoperatively, and the animal demonstrated no signs of distress during the following days of continuous monitoring. Weekly weighing and general assessments confirmed that the animal was thriving throughout the study period. The animal was euthanized after three uneventful weeks in the stables, and the conduit specimen was retrieved for histological evaluation. After removing the inner plug of the conduit, an open lumen leading into the bladder could be inspected, and no macro- or microscopic signs of infection or rejection were observed in the conduit surroundings (Fig. 7c). Macroscopically, the luminal outline of the stent was visible below a continuous epithelium, whereas the micrografts were no longer visible. Positive stains for luminal epithelialization (pancytokeratin AE) were demonstrated in both the proximal, medial, and distal segments of the conduit. In addition, positive stains for urothelial differentiation (uroplakin-II) were demonstrated in all three segments of the conduit (Fig. 7d–f).

## Discussion

In this study, we investigated the optimal conditions for the assemblance of a urinary conduit, which could be constructed and surgically implanted during one continuous procedure. With the primary aim of designing a scaffold suitable for single-staged in vivo implantation, as a bladder conduit, several biological properties had to mimic those of a native urethra. Therefore, various aspects of our construct were assessed simultaneously. By using porcine urothelial tissue micrografts, we concluded that a tubularized version of the collagen scaffold, enforced with commonly available surgical materials, could be easily assembled in the laboratory, and that stratified urothelial cells could be identified on the luminal scaffold side after 4 weeks in culture. Furthermore, the process of in vitro scaffold biodegradation did not affect pH in the culture medium in vitro. After comparing two different collagen densities, we concluded that the higher collagen proportion (80%) provided a better barrier function, with less permeability to large molecules, and that this concentration also demonstrated tendencies of less collagen delamination. Lowering the collagen concentration, furthermore, significantly increased the reduction of the scaffold thickness over time. The positioning of micrografts, either on top or below the collagen layer, did not affect the regenerative potential of the micrografts nor the rate of collagen delamination. As the submerged micrograft positioning intuitively provided better mechanical stability, this construction was applied in our subsequent in vivo experiment. Despite partial collagen delamination in a minority of samples, the urothelial cells would readily colonize the mesh material instead. Moreover, we confirmed that our scaffold provided an equally favorable platform for the proliferation of human urothelial cells, harvested from non-invasive bladder-washings, as well.

Correct application of supporting biomaterials has previously proven pivotal for the biomechanical success of a scaffold^15,18^. From biomechanical assessments, we concluded that the course direction of the knitted mesh material was more suitable for the longitudinal conduit requirements (i.e., due to increased extensibility), whereas the wale mesh direction provided sufficient circumferential strength for the surgical implantation. Upon construction, the scaffold greatly outperformed the strength of a native urethra, whereas the scaffold strength subsided during the 4-week incubation period. This latter finding, however, encouraged us to continue with in vivo scaffold insertion, as one of the key elements to a successful implantation in a clinical setting would indeed require complete biodegradation of the scaffold.

Therefore, in continuation of our in vitro experiments, we concluded that it was feasible to implant our tubular scaffold in a live minipig model, using the higher collagen density and with micrografts submerged within the scaffold. The surgical implantation proved technically manageable as a single-staged procedure and was completed within the normal timeframe of a routine surgery. Three weeks after successful implantation, the animal was well and thriving, and the resected conduit showed favorable regeneration with multilayered urothelium covering the entire lumen throughout the five-centimeter-long conduit.

Previously, skin micrografts have been studied and clinically applied for several decades, however, knowledge on the optimal scaffold composition for application in hollow organs remains scarce^19,20^. Furthermore, the in vivo survival and regeneration of directly implanted urothelial micrografts, circumventing preimplant ex vivo cultivation, remains largely unexplored. In prior studies, we have been successful with both in vitro and in vivo expansion of tissue micrografts^15,21,22^. However, in previous porcine conduit models, we experienced detachment of the neo-regenerated urothelium and lack of basement membrane formation, as a recurrent problem^23–26^. Recently, we have demonstrated how skin micrografts, compressed in a collagen scaffold, survive and regenerate when surgically implanted subcutaneously in live rats, further strengthening our hypothesis of utilizing the body as a bioreactor after a single-staged implantation^27^.

In the currently presented construct, collagen acted as a favorable tissue adhesive, maintaining micrograft attachment to the conduit despite mechanical manipulation. At the same time, the collagen provided a suitable environment for cell migration and proliferation. Whereas collagen scaffolds have previously been studied in various tissue-engineered models, completely submerging micrografts within the collagen has, to our knowledge, not been done before, and represents a potential improvement to the durability of this type of scaffold. Another important feature of the scaffold was the ability to maintain a proper barrier function, in this case between the contents of the urinary bladder and the external environment, until the scaffold has been completely integrated within the organism. One concern, before in vivo implantation, was whether the high-density collagen would restrict the migration of cells and the diffusion of nutrients from the external environment to the luminal micrografts of the conduit. Therefore, to establish an optimal collagen density balancing both these issues, various properties of two different collagen concentrations were compared. In agreement with our previous study^15^, we demonstrated that applying equal force of compression to different concentrations did not lead to significant difference between the final thickness of the two hydrogels (as seen after 2 weeks in culture). This indicates that our aim to get compressed collagen of lower density (60%) was met, however, the significant reduction in thickness after 4 weeks could relate to differences in the collapse of the 3D construct, and we speculate whether full-thickness tissue seeding would avoid this collapse. On the other hand, regarding the importance of tissue impermeability, we continued using the higher collagen density which maintained a superior barrier function. In alternative experiments, the compression weight could be adjusted according to the collagen concentration, and thereby levelling the final collagen density. Finally, from the results of in vivo conduit implantation, we did not observe signs of restrictions in the luminal nutrition of the micrografts during regeneration. Nevertheless, further studies are required to confirm this assumption. In retrospect, the use of deionized water in the permeability test may not have been optimal, as it may risk a non-physiological hypotonicity. In future assays, we would recommend using an isotonic solution. Although the results are, admittedly, based on small-scale experiments, we believe that our findings provide sufficient indications that the basic methodology is feasible for further investigations. From a clinical perspective, our in vivo model did not address the issue of conduit stenosis, as our tubular scaffold was not anastomosed to the skin level. Nonetheless, in this proof-of-concept pilot model, our aim was merely to investigate short-term mucosal regeneration and, furthermore, any severe potential adverse graft-versus-host reactions. Moreover, although we found similar in vitro integration of human urothelial cells within the scaffold, any potential interspecies differences, between man and minipigs, in this setting, can currently only be speculated upon.

Previous preclinical studies, on both urethral and ureteral animal models, have favored the implantation of cell-laden tubular matrices, since acellular scaffolds have demonstrated increased stricture formation^28–32^. Nonetheless, the clinical translation of these techniques is often hampered by the resource-intense requirements related to multi-staged surgeries involving ex vivo cell cultivation. In this context, single-staged autologous micrografting offers a simple and widely reproducible alternative, as the collagen-based scaffold with autologous micrografts allows for constructing and tailoring a conduit during the primary reconstructive surgery. By perioperatively harvesting autologous native bladder mucosa, for the immediate construction and implantation of the conduit, the patient can be spared of several subsequent surgeries. Enforcing the scaffold with biodegradable and commonly available surgical materials (i.e., mesh and stent) ensures adequate robustness for surgical handling. Nevertheless, all elements in the composite graft will need to be approved for human use before introduction to the clinic. Furthermore, the micrografting technique allows the surgeon to expand a small sample of healthy native tissue to a relatively larger scaffold area, which is often required in reconstructive surgery of congenital malformations. Moreover, in a human setting, the single-staged implantation of a tissue-engineered bladder conduit could theoretically be performed within the extraperitoneal space only, hereby sparing the patient of potential intraperitoneal adhesions and subsequent complications hereof.

In conclusion, our current model offers a simple methodology for constructing a viable bladder conduit, and our findings have encouraged us to aim at further exploring the method in preclinical large-scale animal studies. Ultimately, we hope our findings may contribute to the future development of optimized surgical treatment options for patients in need of long-term urinary diversion.

## Methods

### Porcine bladder micrograft harvesting

Urinary bladder mucosa was harvested from three full grown female Landrace pigs, which had been used for non-related extra-abdominal surgical training. Immediately after animal euthanization, the bladders were excised under sterile conditions via midline laparotomy and transported back to the laboratory in vials with 1 × DMEM (Dulbecco’s Modified Eagle Medium, Sigma-Aldrich, St. Louis, US) and antibiotics (penicillin 50 U/mL and streptomycin 50 µg/mL, Invitrogen, Thermo Fisher Scientific, Waltham, US). The fresh organs were cut open and washed in PBS (phosphate buffered saline, Sigma-Aldrich). A segment of the anterior bladder wall was fixated to a cutting board and the mucosa was dissected for careful removal of connective tissue. The mucosa was finally minced to micrografts of approximately 1 mm^2^ with a scalpel and used directly for experimentation. Unless stated otherwise, the following in vitro experiments were based on these three biological replicates, with subsequent technical replicates constructed when appropriate.

### Plastic compressed collagen scaffolds

The collagen-based scaffold was constructed as described in previous publications^16^ . Two different concentrations of collagen solution were tested and compared (60% vs. 80%). In brief, a solution of type I rat tail collagen (2.06 mg/ml protein in 0.6% acetic acid, First Link Ltd, Wolverhampton, UK) was mixed with 10 × MEM (Gibco, Thermo Fisher Scientific, Waltham, US) while kept on ice. Previously, we have used the 80% concentration of collagen solution (1.64 mg/mL collagen in the final hydrogel), in accordance with the majority of previous collagen-scaffold studies, however, in this study we compared with a 60% concentration of collagen solution (1.23 mg/mL collagen in the final hydrogel) as well. The pH was carefully monitored, as droplets of 1 M NaOH (Gibco) was slowly added, until reaching 7.4. The solution was then mixed with 1 × MEM (Gibco) to adjust the final collagen concentration. The liquid solution was poured into a steel mold and incubated in 37 °C for 5 min. After setting in the incubator, a knitted polyglactin mesh (Vicryl™, VM1208, Ethicon, Johnson & Johnson, New Brunswick, US) was placed on top of the newly formed gel, and another layer of fluid collagen solution was poured on top of the construct. After another 5 min in the incubator, the construct was seeded with bladder mucosal micrografts as described above. The entire construct was then temporarily covered by a nylon mesh and compressed with 120 g for 5 min, to expel water from the gel (Fig. 1a). The final collagen concentrations after compression were estimated to 32.8 mg/mL and 24.6 mg/mL, respectively^15^.

### Culture medium

In all of the following in vitro experiments, a customized culture medium consisting of the following components was used: A 4:1 mix of DMEM:Ham’s F12, (Gibco-BRL Technology, UK) supplemented with fetal bovine serum 10% (Gibco-BRL Technology, UK), insulin 5 µg/ml, hydrocortisone 0.4 µg/mL, adenine 21 µg/mL, cholera toxin 10^−10^ mol/L, triiodothyronine 2 × 10^−9^ mol/L, transferrin 5 µg/mL, epidermal growth factor 10 ng/mL (all from Sigma-Aldrich), penicillin 50 U/mL and streptomycin 50 µg/mL (Invitrogen).

### Permeability assay in vitro

The diffusion of bovine serum albumin (Sigma-Aldrich) across scaffolds from each biological replicate with a high collagen concentration (80%) was compared with scaffolds with a lower concentration (60%) at different timepoints after cell seeding. Albumin was chosen due to its relatively large size, and since it serves as an important osmotic regulator in vivo. The collagen construct was mounted in a 1.28 mm unjacketed Franz Diffusion Cell with flat ground joint, 8 ml receptor volume, stirbar, and pinchclamp (SES GmbH Analyse Systeme, Westerwald, DE) and filled with demineralized water. The upper donor chamber was filled with deionized water with added albumin at a concentration of 5 mg/mL, and the lower recipient chamber was filled with deionized water alone. Samples were collected from the recipient chamber at fixed time intervals (1, 30, 60, 120, 180, 240, 300, and 360 min) for photometric analysis at 278 nm using a NanoDrop™ 2000 (Thermo Fisher). A standard curve based on known albumin concentrations was used to estimate the changes in albumin concentration across the collagen scaffold over time, as a measure of changes in permeability.

### Graft degradation in vitro

Collagen scaffolds from each condition (i.e., 60% vs. 80% collagen solution, and with micrografts either on top or submerged within the collagen) were fixed after either 2- or 4 weeks in culture for conventional histology, as described below. To evaluate changes in the scaffold thickness over time, microscopic sample scans, blinded to the assessor, were measured as follows: the middle of the scaffold sample was estimated, and from here, full-wall measurements of scaffold thickness were annotated every 500 µm for 10 consecutive measurements in each lateral direction (Fig. 2e).

### Tubular conduit construction in vitro

In this experiment, a compressed high-density collagen scaffold, with submerged urothelial micrografts from one of the porcine bladders, was rolled around a biodegradable polydioxanone (PDS) stent (SX-ELLA Degradable Biliary DV stent, ELLA-CS, Trebes, CZ) which had been specifically customized for the purpose (length 5 cm, inner diameter 6 mm). The construct was sutured longitudinally with running Vicryl™ 4-0 suture (Ethicon). The tubular scaffold was cultured (37 °C and 5% CO_2_) in a 25 cm^2^ flask with 15 ml keratinocyte medium (described above) which was changed every 24 h (Fig. 1a,c,f). When changing the culture medium, the used medium was analyzed directly using a ABL800 FLEX blood gas analyzer (Radiometer Medical ApS, Copenhagen, DK). After 4 weeks in culture, the conduit was submerged in 4% formalin (Sigma-Aldrich) for 24 h before paraffin embedding and transversal sectioning.

### Biomechanical properties in vitro

The samples were mounted with grasping devices onto a TA.XTplus Texture Analyzer™ (Stable Micro Systems Ltd, Godalming, UK) equipped with a 2N loadcell, and were subjected to uniaxial tensile tests by applying the samples to 10 mm/m strain until complete sample rupture. Since the physical backbone of the scaffold consisted of a knitted Vicryl material, multiple spikes on the stress curve would occur before ultimate sample fracture, as the individual knitted masks would break, and, therefore, a cut-off of a 10% decrease from the maximal measured stress level was used to censor each measurement. The mean and standard deviations were calculated across all replicates in each condition, at each time-dependent measurement, before final comparison across conditions. Tension in either the wale or the course directions of the fresh Vicryl mesh (as illustrated in Fig. 4b), were compared without additional collagen and micrografts, under the assumption that these components would contribute minimally to the biophysical properties. To evaluate the degradation of the construct over time, and the possible influence of co-cultured micrografts, the stress and strain levels were assessed after 1, 2 and 4 weeks in culture.

Finally, the biomechanical properties of a freshly constructed collagen-based conduit, reinforced with Vicryl mesh and sutured as described above but without an indwelling PDS stent, was compared to a fresh porcine urethra. Uniaxial tensile tests were done both in the longitudinal (course) direction and in the circumferential (wale) direction, the latter by using custom made hooks (Fig. 4f,h).

### Bladder wash cell harvesting and cultivation in vitro

As an alternative to adding porcine bladder micrografts to the collagen scaffold, a cell-suspension of in vitro propagated human urothelial cells were seeded on top of the construct. These cells were obtained as previously described by washing the urinary bladder with saline in a patient undergoing catheterization during a routine hypospadias surgery^33^. The bladder was filled with 50 ml sterile isotonic saline five times and the barbotage (approximately 250 ml) was transported directly to the laboratory (Fig. 5a). Here, 50 ml vials containing the bladder wash were centrifugated for 5 min at 1000×*g* and the supernatant was then removed. After resuspending with DMEM (2 ml in each vial), the cell-suspensions were pooled and centrifugated once more at 1000 rpm for 5 min. The final pellet was resuspended in 2 mL of keratinocyte culture medium and plated in a 6-well culture plate (one well) which had previously been coated with poly-l-lysine. When cell colonies had reached approximately 70–80% confluency (after about 1 week), the cells were trypsinated and transferred to 25 cm2 culture flask for further propagation (Fig. 5b). When a sufficient total number of cells had been reached, approximately 40.000 cells/cm^2^ were seeded on top of each of the plastic compressed collagen scaffolds, and the samples were then kept in cell culture conditions for either 2 or 4 weeks (Fig. 5c). Finally, the samples were fixed with 4% formalin and prepared for histological sectioning as described below. For comparison, a full-wall section of a porcine bladder was used as a reference to native urothelial layering.

### Surgical conduit implantation in vivo

A full-grown female Göttingen minipig (12 months old, weighing 36 kg) was sedated with a mix of Zoletil^®^ (3.6 mg, Virbac, Kolding, DK) and ketamine (125 mg). After endotracheal intubation, anesthesia was maintained with propofol (10 mg/kg/hr) and fentanyl (10 µg/kg/hr). The animal was placed in the supine position and the abdomen was shaved and scrubbed, before washing with 70% ethanol two times and covering with surgical draping. A urinary catheter was placed, and the bladder was filled with 250 mL prewarmed sterile isotonic saline. The urinary bladder was mobilized via lower midline laparotomy, and a 2 × 5 cm full-wall segment of the anterior bladder wall was resected during careful hemostasis. The excised specimen was transferred to another sterile table in the operating room, and 2 × 1 cm of the mucosa was dissected and minced in 1 mm^2^ micrografts (the remaining 2 × 4 cm of excised tissue was used for other research purposes). Subsequently, a mesh-enforced collagen-based scaffold was prepared, in an identical fashion as described above, using an 80% collagen mix and with the micrografts seeded directly on the mesh (submerged below the collagen) at a 1:6 expansion ratio (i.e., 2 cm^2^ of tissue was equally distributed on a 12 cm^2^ mesh). After plastic compression, the construct was sutured around the stent with a running PDS 5-0 suture (Ethicon). Simultaneously, the bladder was closed with running Vicryl™ 2-0 suture, leaving a 1 cm wide proximal opening. The conduit was transferred back to the surgical table and anastomosed to the bladder with running PDS 5-0 suture. Meanwhile, a 14fr ACE stopper (Aquaflush^®^, Abena, Taastrup, DK) was placed within the conduit, to keep open the lumen, and a PDS 5-0 ligature was fixated around the distal end of the conduit for patency. A peritoneal flap from the pubovesical ligament was harvested and used to patch the entire construct including the anastomosis. The conduit was pulled through an adapted opening in the abdominal fascia and fixated in the subcutaneous space, and two interrupted Prolene 3-0 sutures (Ethicon) were tied to the ACE stopper cap and fixated externally in the skin (to fixate and mark the location of the conduit) (Fig. 6a). The abdominal muscle fascia was closed with running PDS 2-0 (Ethicon) and the subcutis was adapted with interrupted Vicryl 4-0 (Ethicon). Finally, the skin was closed with running Prolene 3-0 (Ethicon), the anesthetic infusion was halted, and the animal was extubated. The animal was treated with buprenorphine (0.1 mg/kg/8 h intravenously) for the first three postoperative days, meloxicam (0.4 mg/kg/day orally) for the first four days, and trimethoprim (96 mg/day orally) and sulfadoxin (480 mg/day orally) for the first five days. After three uneventful weeks of single housing in the stables, the animal was terminated with pentobarbital injection (100 mg/kg intravenously). The conduit was excised directly post-mortem and fixed in 4% formalin for 24 h. Transverse sections of the proximal, medial, and distal conduit were prepared for histology and stained with H&E, CK-AE, and UPII as described below (Fig. 6b–f). As this was an exploratory pilot study, evaluating the technical feasibility of the procedure, no control groups were included. This in vivo animal pilot experiment was conducted and reported in accordance with the ARRIVE guidelines (checklist submitted to the journal separately)^34^.

### Histology

The samples were fixed in buffered 4% formaldehyde for at least 24 h, then dehydrated and embedded in paraffin, before 3 μm microtome sectioning. After rehydration, staining with either hematoxylin–eosin (H&E) or Masson’s trichrome (MTC) was performed for routine histology. In addition, the following immunohistochemical antibody stains were used: pancytokeratin CK-AE (Clone AE1/AE3, ID: GA053, DAKO Agilent, US), collagen IV (clone CIV 22, ID: 760-2632, Roche, CH), ki67 (clone MiB-1, ID: GA626, Agilent, US), uroplakin II (rabbit monoclonal ERP18799, Abcam, Cambridge, UK). Sections were scanned with the Visiopharm Oncotopix scaner (Hamamatsu, Shizuoka, JP).

### Scanning electron microscopy (SEM)

Samples were fixed in 4% formaldehyde for 24 h and dehydrated with increasing concentrations of ethanol (30%, 50%, 70%, 90%, and 100%) for 10 min each and then treated with hexamethyldisilane (Sigma-Aldrich) overnight for further water extraction. Dehydrated samples were then mounted on aluminium stubs with double sided carbon tape (Agar Scientific, Essex, UK), and imaged with a tabletop scanning electron microscope (TM3030Plus, Hitachi Hightech, Tokyo, JP) at appropriate magnifications.

### Statistical analyses

Unless otherwise stated, numbers are presented in means with standard deviations (SD). For direct comparison of continuous variables, independent two-tailed t-tests were applied with *p* < 0.05 considered as statistically significant. Data was analyzed using Microsoft Excel 2016 (Microsoft Corporation, Washington, US) and RStudio (version 2022.07.1 build 554, R Core Team 2020, Vienna, AUS).

### Ethical permissions

For the in vitro experiments, porcine tissues were harvested post-mortem after unrelated surgical training, and, therefore, no ethical permissions were required for this part of the study. Harvesting of urothelial cells from patient bladder wash was performed after informed consent, and in accordance to ethical permission granted by the Danish Ministry of Health (ref. no. H-20084445). The in vivo animal experiment was performed in accordance with an ethical permission granted by the Danish Ministry of Food and Agriculture (Ref. no. 2022-15-0201-01206).

## Data Availability

Any data not presented in the publication can be made available upon request directly to the corresponding author.
